# Determinants of Parental Purchase Intent for Functional Shoes for the Growing Feet of Preschool Children: A Cross-Sectional Study in Japan

**DOI:** 10.7759/cureus.90856

**Published:** 2025-08-24

**Authors:** Misako Uchita, Masahiro Takemura, Jun Sugawara

**Affiliations:** 1 Sports Medicine, Graduate School of Comprehensive Human Sciences, University of Tsukuba, Tsukuba, JPN; 2 Department of Nursing, Chronic Care Nursing, and Diabetes Nursing, Tsuchiura Kyodo General Hospital, Tsuchiura, JPN; 3 Institute of Health and Sport Sciences, University of Tsukuba, Tsukuba, JPN; 4 Human Informatics and Interaction Research Institute, National Institute of Advanced Industrial Science and Technology (AIST) Faculty of Health and Sport Sciences, University of Tsukuba, Tsukuba, JPN

**Keywords:** children's shoes, cross-sectional study, foot development, general consciousness, health education, health promotion, japanese people, prefectures, preschool children, preventive medicine

## Abstract

Background

At birth, children's feet are immature both structurally and functionally. Selecting functional shoes is thus vitally important for their healthy feet and gait development during the growing stage. In some Western countries, podiatrists, among other specialists, provide clear foot evaluations and health guidance on selecting the best functional shoes for children from early childhood. In Japan, however, there are no similar, well-established systems, and many parents select shoes without professional support. This study aims to identify factors influencing purchase intent among parents who, after receiving video-based information about functional shoes in different development stages, recognized the need but still responded that they would not or could not purchase such shoes. The Health Promotion Model (HPM) was adopted as the theoretical framework for analysis.

Methodology

This cross-sectional study was conducted online as a survey in Google Forms. Participation was completely voluntary. Only parents who provided informed consent after going through the outline of the study were included. The participants were parents of preschool children in the childcare facilities operated by the Ibaraki Prefectural Childcare Council in Japan. The survey covered personal characteristics, perceptions of the necessity of functional shoes, and factors influencing purchase intent.

Results

A total of 4,097 questionnaires were distributed, and 448 (10.9%) responses were collected. Of these, 39 (8.7%) respondents who answered that functional shoes were “not necessary” were excluded, and the remaining 409 (91.3%) respondents who recognized the necessity were included in the analysis. Among the 409 respondents (91.3%), 354 (86.6%) were female, 51 (12.5%) were male, and four (1.0%) did not report their gender. In terms of age distribution, 360 respondents (88.0%) were in their 30s or 40s. Among the 409 respondents (91.3%), 221 (49.3%) stated that they “wanted to purchase” functional shoes, while 188 (42.0%) answered that they “would not or could not purchase."

Four determinants were then identified: (1) support and influence from professionals, family, and the social environment; (2) physical characteristics and concerns about the child's growing feet; (3) knowledge and understanding of foot development and functional shoes, and (4) perceived effectiveness of functional shoes and expectations for support. The impact of factors (1), (3), and (4) on purchase intent was found to be significant. Moreover, household income was also closely correlated.

Conclusion

This study revealed that a lack of knowledge regarding functional shoes and an insufficient understanding of children’s foot development are determining factors influencing parental purchase intent. In addition, household income was found to be closely related to purchase intent. Furthermore, purchase intent might not be followed by an action due to family issues or the absence of support, even when parents recognize the importance of functional shoes. To facilitate footwear selection and purchase, it is essential to enhance the provision of support from professionals and the establishment of a sound support system for the public.

## Introduction

The human foot has a complex structure adapted for bipedal locomotion. Its functionality relies on the coordinated development of the skeletal system and soft tissues such as muscles, tendons, and ligaments, enabling both flexibility and stability [1]. When we walk, the rearfoot, midfoot, and forefoot function as distinct segments with specific roles, working together to provide both stability and propulsion [2]. In early childhood, however, the bones are not fully ossified, the ligaments are soft, and the foot arches are not yet formed, so the structural and functional development of the foot is not yet completed at this stage [3]. Between the ages of three and six, the medial and transverse arches begin to develop, and children often present with physiological genu valgum (knock-knees) and rearfoot overpronation [4,5]. While the flatfoot tendency associated with rearfoot overpronation may improve by itself as the child grows, factors such as obesity, ligamentous laxity, and genetic predispositions may get into the process [6]. Flat feet have been identified as a risk factor for increased stress on soft tissues, lower limb disorders, and impaired walking function [7,8]. In particular, overpronated feet have been reported to adversely affect load distribution and propulsion during gait [9], making rearfoot alignment control a key developmental concern. One of the clinical indicators used to assess this is the leg heel angle (LHA), which visualizes rearfoot valgus and represents a critical aspect to monitor during growth, highlighting the importance of ensuring appropriate support for the rearfoot. Therefore, functional shoes with specially designed structures to support the rearfoot according to the developmental stage are considered crucial.

Meanwhile, the functionality and effectiveness of children's shoes remain debated due to the lack of valid evidence and solid guidelines [10-12]. Nonetheless, in practice, children aged three to seven years with genu valgum often present with rearfoot valgus. Reflecting this, the Japanese Society of Footcare and Podiatric Medicine has issued a clinical recommendation suggesting the use of supportive high-cut shoes during this developmental stage, based on the usage standards outlined in Germany's WMS (Weitenmaßsystem) guidelines [13]. This guideline also notes that the culture of functional shoes from Germany, a leading country in footwear, was introduced to Japan about 30 years ago, but has not become widespread. In Japan, cultural and economic factors, such as a tendency to prefer shoes that can be easily put on and taken off without using hands, and a greater expenditure on children’s clothing than on footwear, may have influenced parental purchase intent. Furthermore, opportunities to learn about children's foot development are limited, and educational or institutional systems to support parental decision-making are not yet well established.

For this reason, the dissemination of such recommendations alone may not be sufficient to shift parental awareness into concrete purchasing behavior. Despite the presence of clinical guidance, parents may face emotional, financial, or contextual barriers, suggesting the need for a more comprehensive support system that facilitates decision-making and behavioral change.

A range of factors may influence parental purchase intent, such as concern for the child’s health, economic status, parenting environment, and access to information. As noted above, parents have limited opportunities to learn about foot development, and many may, under the influence of multiple factors, choose easier and more readily available options without considering any functional features. This situation is reflected in the Health Promotion Model (HPM), proposed by Nola J. Pender, which provides a theoretical framework for explaining the process by which individuals engage in health-promoting behaviors [14]. The HPM consists of three major components: individual characteristics and experiences, behavior-specific cognitions and affect, and behavioral outcomes.

This study aimed to identify the determinants of parental purchase intent for functional shoes for the growing feet of preschool children, applying the HPM as a comprehensive framework to explain how multiple interacting factors shape health-related decision-making.

## Materials and methods

Study design

From November to December 2024, we conducted an online self-administered survey targeting parents of preschool children attending childcare facilities in Ibaraki Prefecture. Cooperation was obtained from multiple facilities belonging to the Ibaraki Prefectural Childcare Council. Along with distributing a document explaining the purpose and significance of the study, we informed participants that participation was voluntary, that non-participation would not result in any disadvantage, and that submission of the completed questionnaire would be considered as consent to participate. The survey was conducted anonymously, and no personally identifiable information was collected. Respondents were informed that they could not withdraw their responses after submission.

Ethics approval

This study was approved by the Ethics Committee of the University of Tsukuba (approval no. TAI 023-141) and conducted in accordance with the Declaration of Helsinki.

Setting and regional context

The target area of Ibaraki Prefecture is located in the northeastern part of the Kanto region of Japan, where urban and rural areas coexist. The prefecture is divided into five regions - northern, central, southern, western, and eastern - each with distinct industrial structures and educational characteristics. For example, the northern region is home to Hitachi City, known globally as a hub of electronics manufacturing and heavy industry. The southern region includes Tsukuba City, where major national research institutions such as the Japan Aerospace Exploration Agency (JAXA) are located.

In rural areas, declining birth rates have led to school consolidations, and long commuting distances may adversely affect children’s quality of life. Given these potential differences in educational and economic backgrounds, our analysis incorporated regional distinctions to examine differences in parents’ purchase intent.

Inclusion Criteria

Parents or guardians who provided informed consent and submitted the online survey via the designated platform were included.

Exclusion Criteria

Participants who did not complete all items and those who responded that functional shoes were “not necessary” were excluded.

Sampling method and sample size

The survey items designed to identify factors influencing purchase intent were developed based on the HPM proposed by Pender, which provides a comprehensive framework for explaining factors related to health-promoting behaviors [14]. The content validity was evaluated by a panel of 10 HPM experts using the Content Validity Index (CVI) [15]. As a result, a final set of 57 questionnaire items was developed (see Appendix). The survey was conducted via Google Forms (Google LLC, Mountain View, CA, USA).

Data collection instruments and procedure

Questionnaires were mailed to 46 cooperating childcare facilities in Ibaraki Prefecture (about 9% of the total 511 facilities). Facility directors then distributed them to parents. Each packet included a QR code linked to an educational video on children’s foot development and a URL to the online questionnaire. The video, created by the authors based on the “Children’s Footwear Guidebook” [16] published by the Japanese Society of Footcare and Podiatric Medicine and referencing Germany’s WMS standards, was confirmed to have been viewed by the respondent before proceeding with the survey.

Demographic information (sex, age, household income, residential area), perceptions of the necessity of functional shoes, and purchase intent were collected. Parents who answered that functional shoes were “necessary” were asked about their intent to purchase. Responses were rated using a five-point Likert scale from “strongly agree” to “strongly disagree.” Factor analysis determined that at least five responses per item were needed, requiring approximately 285 participants. Considering an average web survey response rate of 10-30% in Japan and the exclusion criteria, the survey was closed when 4,097 responses were collected.

The questionnaire used in this study was developed based on relevant literature and expert review [17], and the full text is provided as supplementary material.

Statistical analysis

We used Google Forms’ filtering function to automatically exclude respondents who answered that functional shoes were “not necessary.” Participation characteristics were summarized using descriptive statistics and percentages, and quantitative variables were presented as means and standard deviations (SD).

Factor analysis was performed on the 57 items using the maximum likelihood method with Promax rotation. Four factors were extracted at the point where the eigenvalue change plateaued, as determined by a scree plot [18], and the internal consistency of each factor was confirmed using Cronbach’s alpha.

Factor scores for each participant were then calculated and used in subsequent analyses. One-way analysis of variance (one-way ANOVA) was conducted to examine differences in factor scores by purchase-intent group, with household income included as a covariate. When significant differences were observed, Bonferroni’s post-hoc test was performed [19].

All statistical analyses were conducted using IBM SPSS Statistics version 29.0 (IBM Corp., Armonk, NY, USA), with a significance level set at 5%.

## Results

Overview of the respondents

In total, 4,097 questionnaires were distributed, and 448 (10.9%) valid responses were collected. Among them, 409 (91.3%) respondents expressed that functional shoes were “necessary,” and 39 (8.7%) who answered that they were “not necessary” were excluded from the analysis.

**Figure 1:**
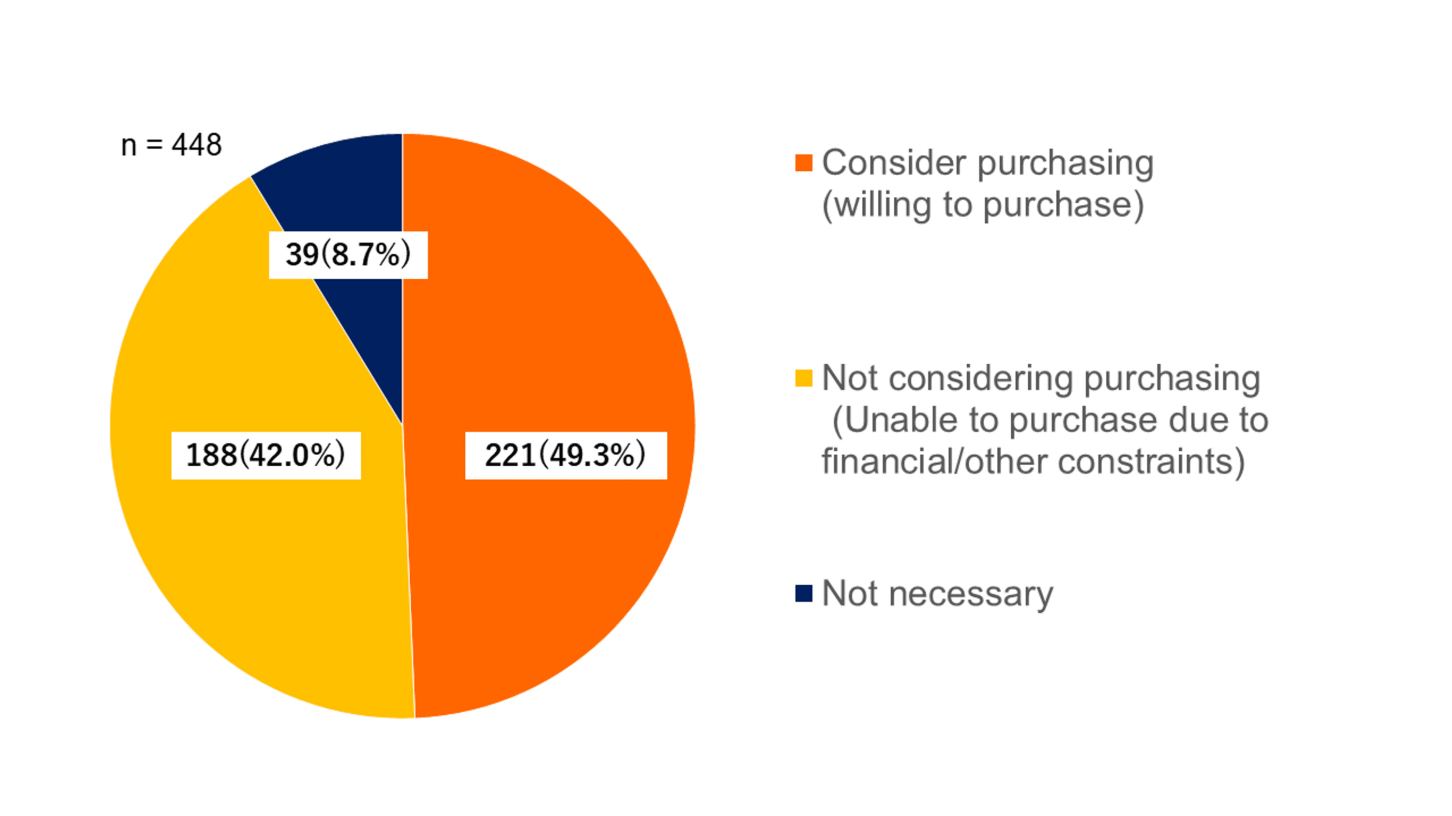
Responses regarding the necessity of and intention to purchase functional footwear for children (n = 409). Responses in a multiple-choice question are classified into three groups: (1) Vivid orange - believe that functional footwear is necessary, willing to purchase (n = 221, 49.3%); (2) golden yellow - believe that functional footwear is necessary but not considering (or not able to) purchase (n = 188, 42.0%); and (3) dark navy blue - believe that functional footwear is not necessary (n = 39, 8.7%).

Of the 409 (91.3%) parents who recognized the necessity of functional shoes, 221 (49.3%) responded that they “would like to purchase,” while 188 (42.0%) responded that they “would not” or “could not purchase” them (Figure 1).

Among these 409 (91.3%) parents, 354 (86.6%) were female, 51 (12.5%) were male, and four (1.0%) did not report their gender.

As for age groups, the largest group was in their 30s: 263 (64.3%), followed by 97 (23.7%) in their 40s, 41 (10.0%) in their 20s, and seven (1.7%) aged 50 or older.

Regarding residential region, 65 (15.9%) lived in the Northern region, 113 (27.6%) in the Central region, 44 (10.8%) in the Western region, 120 (29.3%) in the Southern region, and 63 (15.4%) in the Eastern region.

Household income distribution was as follows: less than JPY four million, 58 (14.2%); JPY four to six million, 121 (29.6%); JPY six to eight million, 118 (28.9%); JPY eight to 10 million, 67 (16.4%); and JPY 10 million or more, 45 (11.0%) (Table 1).

**Table 1:** Demographic characteristics of respondents (guardians) (n = 409) who recognized that functional footwear is essential. Presented in both actual numbers and percentages (n (%)). This table summarizes the demography of guardians who recognized that functional footwear is essential, along with their gender, age group, residential region, and household income.

Demographic characteristics	Category	n (%)
Gender	Female	354 (86.6%)
	Male	51 (12.5%)
	No response	4 ( 1.0%)
Age	20s	41 (10.0%)
	30s	263 (64.3%)
	40s	97 (23.7%)
	50 or older	7 ( 1.7%)
	No response	1 ( 0.2%)
Residential region	Northern area	65 (15.9%)
	Central area	113 (27.6%)
	Western area	44 (10.8%)
	Southern area	120 (29.3%)
	Eastern area	63 (15.4%)
	No response	4 ( 1.0%)
Household income	Less than ¥4,000,000	58 (14.2%)
	¥4,000,000 – < ¥6,000,000	121 (29.6%)
	¥6,000,000 – < ¥8,000,000	118 (28.9%)
	¥8,000,000 – < ¥10,000,000	67 (16.4%)
	¥10,000,000 or more	45 (11.0%)

Results of factor analysis and interpretation of the factor structure

The results of the factor analysis are presented in Table 2.

**Table 2:** Factor analysis results for determinants influencing parental purchase intentions in Ibaraki Prefecture, Japan Four factors were extracted using maximum likelihood estimation with Promax rotation and Kaiser normalization. Items with factor loadings ≥0.40 were included in the respective factor. For clarity, additional spacing was inserted between the list of items and the eigenvalues. Cronbach’s alpha coefficients for the four factors ranged from 0.928 to 0.975. In this table legend, only factor loadings ≥0.40 are shown in bold for emphasis.

	Factor loading			
	Factor 1	Factor 2	Factor 3	Factor 4
[Factor 1] Support and influence from professionals, family, and the social environment regarding functional shoes (Cronbach’s α = 0.975)				
I have specific recommendations from a professional midwife.	1.032	-0.040	-0.066	-0.088
I have specific recommendations from my kid's nursery school teacher.	0.991	-0.028	-0.013	-0.081
I have specific recommendations from a close friend of mine.	0.968	-0.034	-0.080	-0.076
I have specific recommendations from a foot-care nurse.	0.951	0.035	-0.052	-0.031
I have specific recommendations from a children's shoe-fitting specialist.	0.936	0.023	-0.033	-0.056
I have specific recommendations from my parents.	0.910	0.011	-0.082	-0.091
I have specific recommendations from a pediatrician.	0.897	0.049	-0.081	0.056
I have specific recommendations from an orthopedic doctor.	0.896	0.046	-0.043	0.030
Seminars that promote healthy children's foot development are available at childcare facilities.	0.848	0.012	0.045	-0.039
I saw posts/footage on social media to acquire knowledge about functional footwear.	0.842	-0.004	0.010	-0.063
I have specific recommendations from my in-laws.	0.830	0.053	-0.104	-0.056
The local government organizes seminars that promote healthy children's foot development.	0.815	-0.010	0.020	-0.007
Seminars that promote healthy children's foot development are available at hospitals.	0.813	0.010	0.012	-0.014
There is a community nearby that promotes healthy children's foot development.	0.789	0.050	0.061	-0.086
Stores with certified children's shoe fitting specialists/ advisors display a mark so they are easily identifiable.	0.621	-0.106	0.149	0.184
My kid's nursery institute/school has specified footwear.	0.613	0.165	-0.051	0.117
The store where I can buy functional footwear is easily accessible.	0.555	-0.102	0.136	0.163
The stores where I can buy functional footwear are within reach from home.	0.554	-0.044	0.114	0.113
Stores that sell appropriate functional footwear display a certification mark that makes them easily identifiable.	0.524	-0.049	0.115	0.290
It takes a long time to reach a store that sells functional footwear.	0.444	-0.097	0.152	0.218
Functional footwear is readily available online, often without requiring foot measurements or gait evaluations.	0.397	0.067	0.083	0.094
I have other priorities over purchasing at shoe specialties for kids.	0.186	0.098	0.045	0.113
[Factor 2] Physical characteristics and concerns regarding the feet (Cronbach’s α = 0.946)				
My child has extreme limb deformities not appropriate for age (serious bandy-legs, knock-knee, or both).	-0.060	0.933	-0.037	0.084
My child has flat feet.	-0.024	0.850	0.007	0.048
My child has calluses or corns on the soles or toes.	0.033	0.815	0.034	-0.001
I have concerns about my child walking.	0.035	0.811	-0.044	0.096
I have a severe lower limb deformity (serious bandy-legs, knock-knee, or both).	-0.018	0.789	0.061	-0.047
I suffer from an inferiority complex for my leg shape.	-0.078	0.777	0.052	-0.010
My child has ingrown toenails.	0.021	0.774	0.054	-0.061
I have aching feet/ knee pain, so I can hardly walk for long.	0.121	0.767	-0.052	-0.015
I have foot fatigue, so I can hardly walk for long.	0.032	0.762	-0.078	0.006
My child sometimes whines for leg pain.	0.048	0.748	-0.029	0.120
I have concerns about my child’s toenail shape.	0.107	0.674	0.014	-0.226
My child usually refuses to walk.	0.045	0.568	-0.074	-0.060
I have flat feet.	0.027	0.559	0.057	0.031
I prefer low-priced, easy-to-clean shoes because kids' shoes get dirty fast.	-0.153	0.301	0.085	0.092
I think it’s okay to buy low-priced, non-functional shoes for my child because children outgrow them quickly.	-0.052	0.255	0.126	0.161
[Factor 3] Knowledge and understanding of foot development and functional footwear (Cronbach’s α = 0.928)				
I understand that children have aging changes in their leg shape.	-0.072	-0.076	0.962	-0.129
I understand that children's soft tissues, such as muscles, tendons, and ligaments, develop over time.	-0.038	-0.063	0.920	-0.014
I understand that children's foot bones are not fully developed at birth, and they ossify by the age of six.	0.013	0.006	0.890	-0.035
I understand that children are born with flat feet and develop their foot arches by around the age of 3 to 4.	0.052	-0.026	0.879	-0.052
I understand that the calcaneus (heel bone) tends to fall over because of its structure.	-0.058	0.093	0.799	-0.002
I understand the three critical functions of children's footwear: support, stability, and flexibility.	-0.039	0.072	0.676	0.091
I have heard the explanation that children's feet are not fully developed at birth.	0.176	0.106	0.493	-0.014
I have heard the explanation about the essential functions of children’s footwear.	0.212	0.189	0.487	-0.025
I understand that footwear is essential for my child's healthy growth.	-0.083	-0.022	0.465	0.140
I have heard the explanation about the structure and functions of the human foot.	0.170	0.224	0.427	0.018
[Factor 4] Perceived effectiveness of functional shoes and expectation for support (Cronbach’s α = 0.940）				
I knew/witnessed/experienced that flat feet can be improved, and foot arches can be better developed.	-0.007	0.006	-0.044	0.997
I knew/witnessed/experienced improvement of falling issues after the period of knock-knee.	0.000	0.016	-0.077	0.994
I knew/witnessed/experienced that functional footwear improves walking.	-0.003	-0.015	-0.017	0.933
I found that functional footwear fits better into the shape of my child’s feet.	0.174	-0.063	0.165	0.575
I noticed improvements in my child’s walking with functional footwear.	0.164	0.087	0.070	0.521
There are subsidies from the central/ local government when purchasing functional footwear.	0.380	0.055	-0.062	0.517
I know where I should go to purchase functional footwear.	0.286	0.200	0.055	0.338
My child likes the design of functional footwear.	0.185	0.080	0.088	0.235
My child can take off and put on shoes.	0.033	0.021	0.015	0.139
I think that functional shoes are expensive (approx. JPY 13,000 - 21,000/pair).	-0.049	0.082	0.059	0.139
Eigenvalue	26.107	4.412	3.187	2.029
Variance explained (%)	45.802	7.740	5.591	3.560
Inter-factor correlation Factor 1	－			
Factor 2	0.651	－		
Factor 3	0.503	0.552	－	
Factor 4	0.703	0.676	0.625	－
Factor extraction method: Maximum likelihood Rotation method: Promax with Kaiser normalization				

Items with factor loadings ≥0.40 were included in the respective factor. For clarity, additional spacing was inserted between the list of items and the eigenvalues in Table 2. Four factors were extracted as influencing parental purchase intent among those who responded that functional shoes were “necessary." The factors were named respectively according to the semantic content of the relevant items. Internal consistency for each factor was evaluated using Cronbach’s alpha coefficients, which ranged from 0.932 to 0.976, indicating high reliability across all factors.

Factor 1: Support and Influence from Professionals, Family, and the Social Environment Regarding Functional Shoes

This factor reflects the influence of recommendations and support from professionals, family members, and the local community who recognize the importance and role of functional shoes. High-loading factors included recommendations from midwives (1.032), nursery school teachers (0.991), close friends (0.968), nurses specializing in foot care (0.951), certified shoe fitters (0.936), grandparents (0.910), pediatricians (0.897), and orthopedic surgeons (0.896). Other influential factors included seminars held at childcare facilities, local governments, or hospitals (0.848-0.813), information on social media (0.842), the presence of community-based foot health programs (0.789), visible certification marks at retail stores (0.621), designated shoes required by schools or childcare centers (0.613), and the accessibility of stores (0.555).

Factor 2: Physical Characteristics and Concerns Regarding the Feet

This factor represents both the physical conditions of the child and the parent, as well as psychological concerns related to them. High-loading items included leg axis abnormalities such as genu varum, genu valgum, and genu recurvatum (0.933), flat feet (0.850), corns or calluses on the soles or toes (0.815), gait abnormalities (0.811), ingrown toenails (0.774), and complaints of lower limb pain (0.748). It also included the parents’ leg deformities (0.789), body image-related concerns (0.777), and difficulty walking due to pain or fatigue in the feet or knees (0.767).

Factor 3: Knowledge and Understanding of Foot Development and Functional Footwear

This factor encompasses basic knowledge of the foot’s growth process and structure, as well as an understanding of the significance of functional shoes. High-loading items are recognition of physiological changes in leg alignment with age (0.962), the development of muscles and ligaments (0.920), the ossification process of foot bones (0.890), the natural course of flat feet (0.879), the structural characteristics of the calcaneus (0.799), and understanding of basic shoe functions such as support, fixation, and flexibility (0.676).

Factor 4: Perceived Effectiveness of Functional Shoes and Expectation for Support

This factor represents both a subjective sense of improvement after using functional shoes and an expectation of financial assistance. High-loading items included perceived improvement in flat feet (0.997), genu valgum (0.994), and walking (0.933), as well as improved fit to foot shape (0.575), gait improvement (0.521), and governmental or municipal subsidies for footwear (0.517).

Factors influencing purchase intent

Parental Purchasing Intentions

Among the 409 respondents (91.3%) who recognized the necessity of functional shoes, a comparison of factor scores was conducted between those who responded that they “would like to purchase” (221 respondents, 49.3%) and those who responded that they “would not or could not purchase” (188 respondents, 42.0%). Of the total 448 respondents, 39 (8.7%) who answered that functional shoes were “not necessary” were excluded from the analysis.

**Figure 2:**
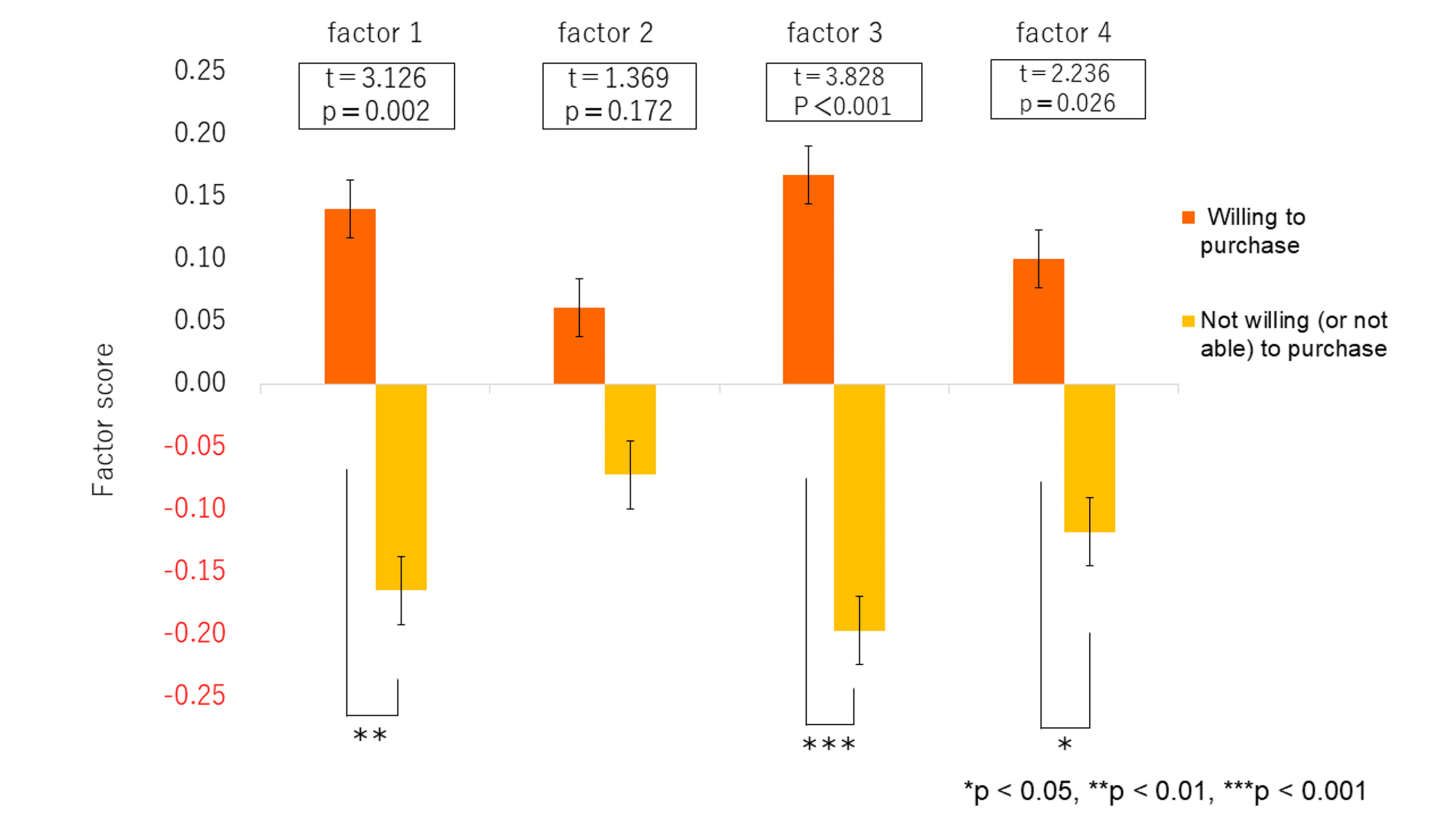
Comparison of factor scores by parental purchase intention for functional (“willing to purchase” or “not willing (or not able) to purchase”) This figure compares the factor scores between guardians who responded that they “consider purchasing” functional footwear (n = 221) and those who “not consider/unable to purchasing)” (n = 188). A t-test was used for the statistical analysis. Data are presented as mean ± standard deviation (Mean ± SD). Significant differences were observed in Factor 1 (t = 3.126, p = 0.002), Factor 3 (t = 3.828, p < 0.001), and Factor 4 (t = 2.236, p = 0.026); no significant difference was found in Factor 2 (t = 1.369, p = 0.172). Statistical significance was set at p < 0.05. Factor 1: Support and influence from professionals, family, and the social environment regarding functional shoes Factor 2: Physical characteristics and concerns regarding the feet Factor 3: Knowledge and understanding of foot development and functional footwear Factor 4: Perceived effectiveness of functional shoes and expectation for support

As illustrated in Figure 2, the “would like to purchase” group had significantly higher scores for Factor 1 (psychological support and social influence), Factor 3 (understanding of foot development and shoe function), and Factor 4 (perceived effectiveness of functional shoes and expectation for support). No significant difference was observed between the two groups for Factor 2 (physical characteristics and concerns about the feet).

Household Income

**Figure 3:**
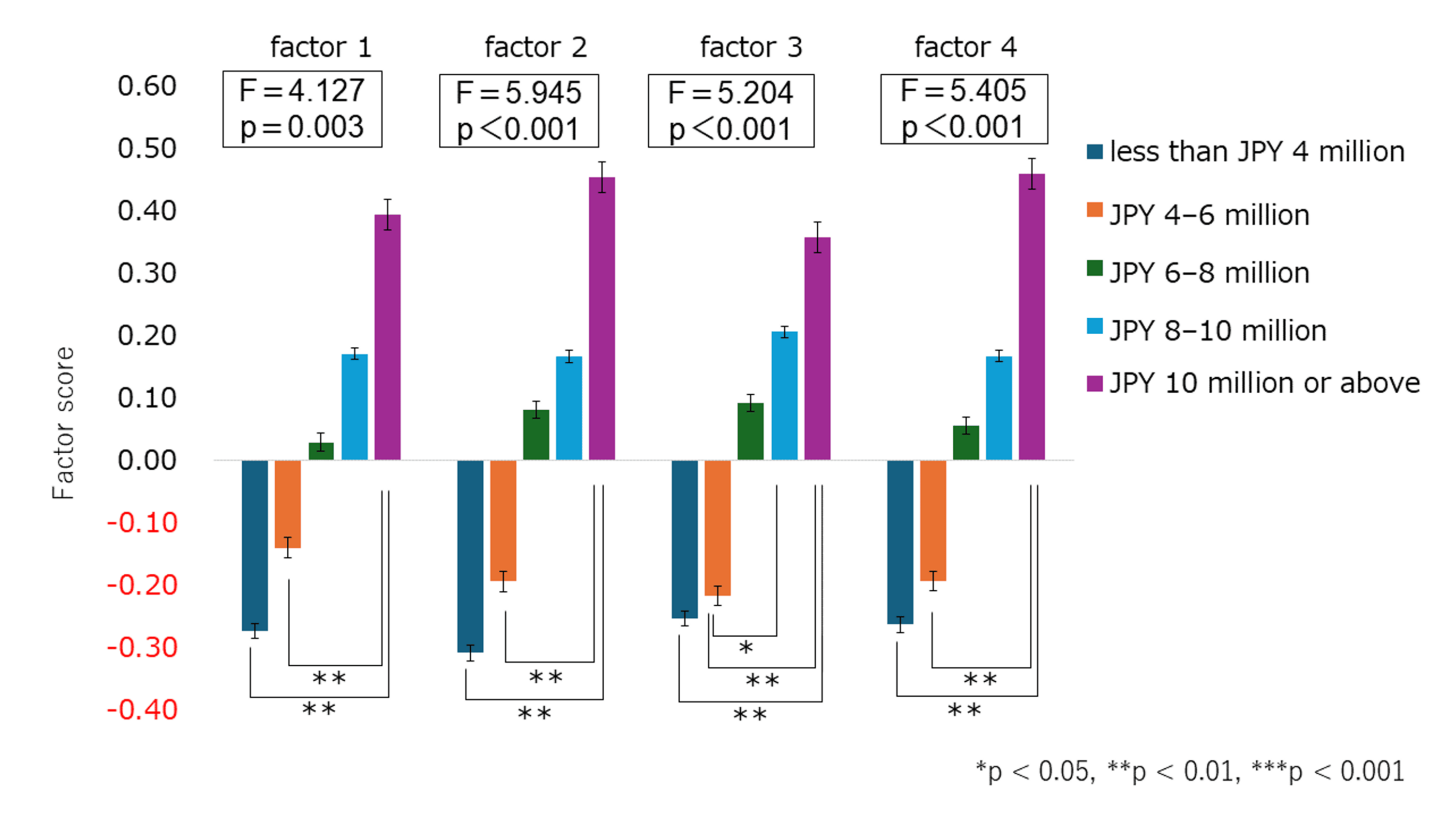
Comparison of factor scores across household income groups This figure compares factor scores between parents who were “considering purchasing” functional shoes (n = 221) and those who were “not considering/could not consider purchasing” them (n = 188). Factor scores were also compared across five household income groups: less than JPY 4 million, JPY 4–6 million, JPY 6–8 million, JPY 8–10 million, and JPY 10 million or above. A t-test was conducted to compare the “considering purchasing” and “not considering / could not consider purchasing” groups. In addition, a one-way ANOVA was conducted across income groups, followed by post-hoc multiple comparisons using the Bonferroni method. Data are presented as mean ± standard deviation (mean ± SD). Significant differences were found in all four factors: Factor 1 (p = 0.003), Factor 2 (p < 0.001), Factor 3 (p < 0.001), and Factor 4 (p < 0.001). Statistical significance was set at p < 0.05, p < 0.01, p < 0.001. Color legend: Dark cyan-blue - less than JPY 4 million, vivid orange - JPY 4–6 million, deep green - JPY 6–8 million, sky blue - JPY 8–10 million, royal purple - JPY 10 million or above

One-way ANOVA was conducted to examine the relationship between household income and each factor score. The results indicated significant differences across all factors, with higher income groups tending to report higher factor scores. Details of the significant differences are presented in Figure 3.

Regional Differences

An analysis was also conducted to investigate whether purchase intent varied by residential region; however, no significant differences were observed. Therefore, regional analysis results were not included in the figures or tables.

## Discussion

This study focused on parents of preschool children who recognized the need for functional shoes but responded that they would not or could not purchase them, for the identification of the factors influencing their purchase intent. The findings suggest that the four factors extracted based on the HPM influence parental purchase intent through diverse aspects, including knowledge, psychological factors, social support, and expectations of functional footwear. Notably, the study addressed the formation and transformation of purchase intent instead of purchase or consumption behaviors. A substantial number of parents recognized the necessity of functional shoes, but still answered that they would not or could not purchase them. This suggests a potential gap between recognition and purchase intent, indicating the need to reconsider how to support those who remain in a transitional stage of behavioral change.

“Knowledge and understanding of foot development and functional footwear” and “Perceived effectiveness and expectation for support” can be interpreted as facilitators that enhance purchase intent among the four determinants identified. By contrast, “Physical characteristics and concerns regarding the feet” functions in two ways: while such concerns may increase awareness, they may also trigger negative emotions or a sense of helplessness, potentially suppressing purchase intent. Similarly, “Support and influence from professionals, family, and the social environment” may boost awareness when reliable sources and specific recommendations are available, but the absence of such support could be demotivating. These findings suggest that some factors may have bidirectional effects depending on the parents’ individual situation or background. Therefore, to effectively support parents who remain at the stage of awareness without taking action, it is essential not only to provide information but also to assess how each factor functions and tailor support accordingly. As shown in Figure 2, factor scores for Factor 1 (Support from professionals, family, and the social environment), Factor 3 (Knowledge and understanding of foot development and functional footwear), and Factor 4 (Perceived effectiveness and expectations for support) were significantly higher among parents who intended to purchase functional footwear compared to those who did not.

Some of the factors identified in this study, such as the provision of knowledge and recommendations from professionals, may already be in practice for certain parents. However, the presence of parents who still report being unwilling or unable to purchase functional shoes despite such information provision suggests that, in some cases, simply receiving information or recommendations may not always be sufficient to shift purchase intent into action. This may highlight the importance of not only the content but also the delivery, timing, and compatibility of support with the recipient’s values and environment. Even when awareness improves, economic constraints, family decision-making dynamics, or personal anxieties may still form barriers to behavioral change. Considering these challenges, future interventions should go beyond one-way information provision and aim to provide phased and interactive support tailored to the diverse circumstances of parents. This approach aligns with Pender’s HPM, which highlights the role of interconnected factors, such as perceived benefits, barriers, self-efficacy, and interpersonal influences, in promoting behavior change, consistent with the interactive nature of the four factors identified in this study. This interpretation is based on the study’s statistical results, the theoretical framework, and practical observations, and aligns with the broader background discussed in this study.

The findings of this study pertain to parental awareness in a specific region (Ibaraki Prefecture), so we have to be careful about data generalization. In Japan, childcare support systems and educational environments differ from one region to another, and not all parents have equal access to professionals or reliable information. Moreover, opportunities to learn about children's foot development, whether at home or in early childhood education settings, remain rather limited. Functional shoes are generally more expensive than standard ones, and economic constraints may significantly influence purchase intent. This is supported by the findings in Figure 3, which demonstrate significant differences in all four psychological and social factors related to purchase motivation across household income groups. These systemic and social factors may serve as a set of hurdles and obstacles when parents choose footwear for their children. Utilizing the findings of this study to inform the development of practical problem-solving strategies is both necessary and timely. Furthermore, these regional disparities are not unique to Ibaraki Prefecture and may reflect broader inequalities in parenting environments between urban and rural areas nationwide, as well as differences in national foot care systems. Future practical interventions must consider these regional and systemic issues and adopt a multilayered, flexible approach that includes not only educational support for individuals but also measures involving childcare facilities, local communities, and administrative systems. Comparative studies involving other prefectures or countries will also be necessary to facilitate learning and progress.

Internationally, there is growing awareness of foot health, and in some Western countries, such as Germany, systems for foot evaluations and footwear guidance by professionals during early childhood have been established. For example, Germany’s WMS (Weitenmaßsystem) promotes footwear recommendations based on foot development stages, with clear standards outlined for health professionals and manufacturers [13]. These practices offer valuable insights, particularly regarding the quality and timing of information delivery to parents.

By contrast, Japan has lagged due to the lack of similar supporting systems. For example, some educational organizations initiated independent programs to promote foot health (“ashi-iku”) among school-aged children, such as those led by the Japan Society for the Study of School Physical Education (JASPE). Nevertheless, systematic support for foot development in both educational and domestic settings remains limited.

When designing future support systems to influence parental purchase intent and behavior, it will be essential to incorporate lessons from international practice while also developing phased and sustainable approaches tailored to Japan’s local childcare environment and policy context.

Limitations

This study was conducted as a cross-sectional online survey and has several limitations. First, due to the nature of the survey method, not all parents may have had equal access to digital devices or stable internet environments. Differences in online access and digital literacy may have introduced selection bias. As the survey was conducted after participants had watched the educational video, the generalizability of the results may be limited. Second, although no statistically significant regional differences were observed in the current analysis, this study was originally designed based on the assumption that regional differences in educational, economic, and social infrastructure might influence parental purchase intent. Contextual factors such as local policies and support systems were not directly measured, and a slightly higher response rate from the Southern area may have influenced the balance of the dataset. Thus, potential regional influences remain a limitation. The response rate in this study (10.9%) was comparable to that of other municipal-level web-based surveys in Japan [20], although it is possible that parents already interested in the topic were more likely to participate voluntarily, which may have introduced selection bias. Third, as this study relied on self-administered questionnaires, respondents might provide socially desirable answers (social desirability bias) unconsciously. In addition, because of the cross-sectional design, the study is not suitable for drawing causal inferences. Further longitudinal research is needed to explore the causal relationships among the influencing factors and the transition from awareness to behavior.

## Conclusions

This study focused on the importance of external support for foot development in preschool children and identified factors influencing parental purchase intent for functional shoes. The results demonstrated that a lack of knowledge about functional shoes and an insufficient understanding of foot development were determinants affecting purchase intent. In addition, this aligns with the HPM’s emphasis on multi-faceted, interactive approaches tailored to diverse parental situations, and it is suggested that, even when awareness changes, action may not follow depending on the environment and availability of support. Therefore, both support for parents and social and environmental support should be considered in future practice and policy development.

## Appendices

**Table 3:** Questionnaire items (English version) This 57-item questionnaire was developed specifically for this study, based on a review of related literature and expert input, following the framework of Pender’s Health Promotion Model (1996).

The English translation of the survey questions of this study	
1	I have heard the explanation about the structure and functions of the human foot.
2	I have heard the explanation about the essential functions of children’s footwear.
3	I have heard the explanation that children's feet are not fully developed at birth.
4	I have flat feet.
5	I have aching feet/ knee pain, so I can hardly walk for long.
6	I have foot fatigue, so I can hardly walk for long.
7	I have a severe lower limb deformity (serious bandy-legs, knock-knee, or both)
8	My child has flat feet.
9	My child has extreme limb deformities not appropriate for age (serious bandy-legs, knock-knee, or both).
10	I have concerns about my child’s toenail shape.
11	My child has ingrown toenails.
12	My child has calluses or corns on the soles or toes.
13	I suffer from an inferiority complex about my leg shape.
14	My child sometimes whines about leg pain.
15	I have concerns about my child walking.
16	My child usually refuses to walk.
17	I understand that footwear is essential for my child's healthy growth.
18	I understand the three critical functions of children's footwear: support, stability, and flexibility.
19	I understand that children's foot bones are not fully developed at birth, and they ossify by the age of six.
20	I understand that children are born with flat feet and develop their foot arches by around the age of 3 to 4.
21	I understand that children's soft tissues, such as muscles, tendons, and ligaments, develop over time.
22	I understand that children have aging changes in their leg shape.
23	I understand that the calcaneus (heel bone) tends to fall over because of its structure.
24	I think that functional shoes are expensive (approx. JPY 13,000 - 21,000/pair).
25	I think it’s okay to buy low-priced, non-functional shoes for my child because children outgrow them quickly.
26	I prefer low-priced, easy-to-clean shoes because kids' shoes get dirty fast.
27	My child likes the design of functional footwear.
28	My child can take off and put on shoes.
29	I noticed improvements in my child’s walking with functional footwear.
30	I found that functional footwear fits better into the shape of my child’s feet.
31	I knew/witnessed/experienced that functional footwear improves walking.
32	I knew/witnessed/experienced improvement of falling issues after the period of knock-knee.
33	I knew/witnessed/experienced that flat feet can be improved, and foot arches can be better developed.
34	There are subsidies from the central/ local government when purchasing functional footwear.
35	I know where I should go to purchase functional footwear.
36	Stores that sell appropriate functional footwear display a certification mark that makes them easily identifiable.
37	Stores with certified children's shoe fitting specialists/ advisors display a mark so they are easily identifiable.
38	The stores where I can buy functional footwear are within reach of home.
39	It takes a long time to reach a store that sells functional footwear.
40	The store where I can buy functional footwear is easily accessible.
41	Functional footwear is readily available online, often without requiring foot measurements or gait evaluations.
42	I have other priorities over purchasing at shoe specialties for kids.
43	I have specific recommendations from a pediatrician.
44	I have specific recommendations from an orthopedic doctor.
45	I have specific recommendations from a foot-care nurse.
46	I have specific recommendations from a professional midwife.
47	I have specific recommendations from my kid's nursery school teacher.
48	I have specific recommendations from a children's shoe fitting specialist.
49	I have specific recommendations from my parents.
50	I have specific recommendations from my in-laws.
51	I have specific recommendations from a close friend of mine.
52	I saw posts/footage on social media to acquire knowledge about functional footwear.
53	My kid's nursery institute/school has specified footwear.
54	The local government organizes seminars that promote healthy children's foot development.
55	Seminars that promote healthy children's foot development are available at hospitals.
56	Seminars that promote healthy children's foot development are available at childcare facilities.
57	There is a community nearby that promotes healthy children's foot development.
